# Selenium-enriched plant foods: Selenium accumulation, speciation, and health functionality

**DOI:** 10.3389/fnut.2022.962312

**Published:** 2023-02-06

**Authors:** Pipat Tangjaidee, Peter Swedlund, Jiqian Xiang, Hongqing Yin, Siew Young Quek

**Affiliations:** ^1^Food Science, School of Chemical Sciences, University of Auckland, Auckland, New Zealand; ^2^Enshi Autonomous Prefecture Academy of Agriculture Sciences, Enshi, Hubei, China; ^3^Riddet Institute New Zealand Centre of Research Excellence in Food, Palmerston North, New Zealand

**Keywords:** selenium, bioactivity, selenium-enriched, functional food, antioxidant, plant food, essential trace mineral, health

## Abstract

Selenium (Se) is an essential element for maintaining human health. The biological effects and toxicity of Se compounds in humans are related to their chemical forms and consumption doses. In general, organic Se species, including selenoamino acids such as selenomethionine (SeMet), selenocystine (SeCys_2_), and Se-methylselenocysteine (MSC), could provide greater bioactivities with less toxicity compared to those inorganics including selenite (Se IV) and selenate (Se VI). Plants are vital sources of organic Se because they can accumulate inorganic Se or metabolites and store them as organic Se forms. Therefore, Se-enriched plants could be applied as human food to reduce deficiency problems and deliver health benefits. This review describes the recent studies on the enrichment of Se-containing plants in particular Se accumulation and speciation, their functional properties related to human health, and future perspectives for developing Se-enriched foods. Generally, Se’s concentration and chemical forms in plants are determined by the accumulation ability of plant species. Brassica family and cereal grains have excessive accumulation capacity and store major organic Se compounds in their cells compared to other plants. The biological properties of Se-enriched plants, including antioxidant, anti-diabetes, and anticancer activities, have significantly presented in both *in vitro* cell culture models and *in vivo* animal assays. Comparatively, fewer human clinical trials are available. Scientific investigations on the functional health properties of Se-enriched edible plants in humans are essential to achieve in-depth information supporting the value of Se-enriched food to humans.

## Introduction

Selenium is an essential trace element for human health. According to the World Health Organization (1), a recommended consumption level of Se is 55-70 μg day^–1^ for adults, with 400 μg day^–1^ as a toxic concentration. Selenium deficiency situation has transpired in some parts of the world, including China (about 72% of the area), Europe (e.g., France and Norway) and New Zealand (2). Selenium is associated with the normal function of glutathione protein (GSH) and its family of antioxidant enzymes such as glutathione peroxidase (GPx), thioredoxin reductase (TrxR) and other selenoproteins (3). The lack of Se can severely affect the human immune system (4, 5), leading to a cardiomyopathy disorder called “Keshan disease” and the bone and joint connection syndrome called “Kashin-Beck disease” (6, 7). Keshan disease occurs when vascular endothelial cells are damaged from oxidative stress due to non-functional antioxidant proteins (8). This disease also causes some serious health problems such as atherosclerosis, hypertension, myocardial necrosis and congestive heart failure (9). Kashin-Beck disease is an endemic osteoarthropathy, causing severe symptoms to joints and bone, including joint pain, elbows flexion and extension disturbances, enlarged inter-phalangeal joints, and limited joint motion (10, 11). Moreover, Se deficiency also increases the risk of arthritis, cancers, and neurodegenerative disorders regarding immune and inflammatory infections (12, 13).

In contrast to Se deficiency, there are a few high soil Se regions globally. The prominent one being the Enshi Province in China, where the soil Se content can rise to 11.4 mg Se kg^–1^ in the high Se area (14). People live in the high Se soil area can suffer from selenosis symptoms and abnormal growth conditions due to excessive Se consumption of foods produced from the area (6, 15). The Se intake of Enshi people was reported to reach 833 μg per day (15), with serum Se concentrations of up to 41.6 μmol L^–1^, approximately 20 times higher than the proposed intake (16). Chronic selenosis is a group of diseases associated with a wide range of symptoms from hair loss, bone and joint problems, and cellular damage from reactive oxygen species which increase the high risk of cancers (17, 18).

In general, toxicity associated with Se intake occurs in a few isolated areas, and food science and technology innovation can help lower Se imbalance intake in the diet. Selenium is present in plant foods in different chemical forms, including the organic Se-containing amino acids, i.e., selenomethionine (SeMet), Se-methylselenocysteine (MeSeCys), and γ-glutamyl-Se-methylselenocysteine (γ-GluMeSeCys), and the inorganic Se, i.e., selenite and selenate (19). Advanced analytical techniques are applied for identifying Se compounds in plant food samples nowadays, contributing to the knowledge of Se chemical forms present in plant foods, their content, and the safe concentration for human consumption. In developing Se-enriched food products, the aim should be focused on providing functional food products to benefit human health and enhance the quality of life. Identification of the Se chemical form and content is essential to justify the use of Se-enriched plant foods for achieving health benefits and overcoming the deficiency issues associated with this essential trace mineral. The objectives of this review are to examine the Se’s accumulation ability and speciation in a wide range of Se-enriched plant foods, to inspect Se and Se compounds’ biological effects on human health, and to explore the prospects of developing Se-enriched plant foods for health purposes.

## Accumulation of selenium in plants

Over the past few decades, Se-enriched plants have been developed to demote deficiency problems for those living in low Se regions who cannot maintain the recommended intake level (18). One of the most simple and robust techniques to increase Se content in plants is by growing plants in high Se soil and applying Se fertilizers. This enrichment method relates to plant species’ absorption, transformation, and accommodation ability of minerals (6). The Se accumulation ability of plants can be classified into three levels: hyper-accumulators, secondary accumulators and non-accumulators. The hyper-accumulators (e.g., *Stanleya, Astragalus*, *Conopsis, Neptunia*, *Xylorhiza*) can accumulate more than 1,000 mg Se kg^–1^ while the secondary accumulators (e.g., *Brassica juncea*, *Brassica napus*, *Broccoli*, *Helianthus*, *Aster*, *Camelina*, *Medicago sativa*) can accumulate between 100-1,000 mg Se kg^–1^. The non-accumulators only accumulate less than 100 mg Se kg^–1^ and most of the angiosperm species are included in this category (20–22).

**FIGURE 1:**
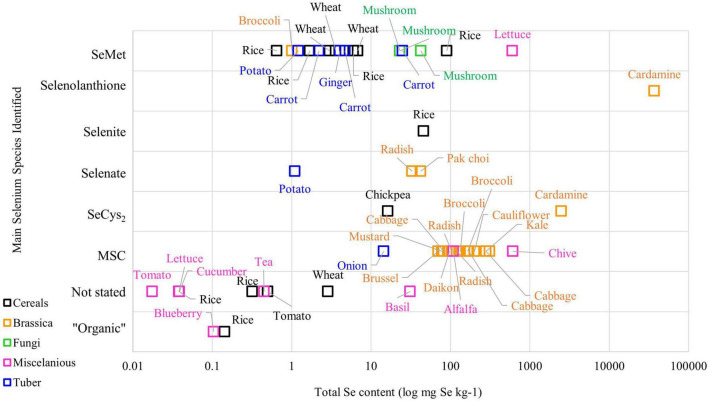
The Se content and chemical species in plant-based foods from the literature (Please refer to Supplementary Table 1 for the original data from the literature).

The metabolism of Se in plant species varies among plants, meaning that different plant varieties can produce different Se chemical forms in various concentrations. Figure 1 demonstrates the complexity of Se chemical forms in different plant species. Literature on Se speciation revealed that the Brassica family, such as broccoli, cabbage, and radish, have MSC as the main Se compound stored in their cells, while SeMet is the main Se chemical form found in cereals grains and tuber crops such as ginger, wheat, and carrot (23–25). On the other hand, selenolanthionine is a major water-soluble Se compound found in *Cardamine violifolia* (26).

**FIGURE 2:**
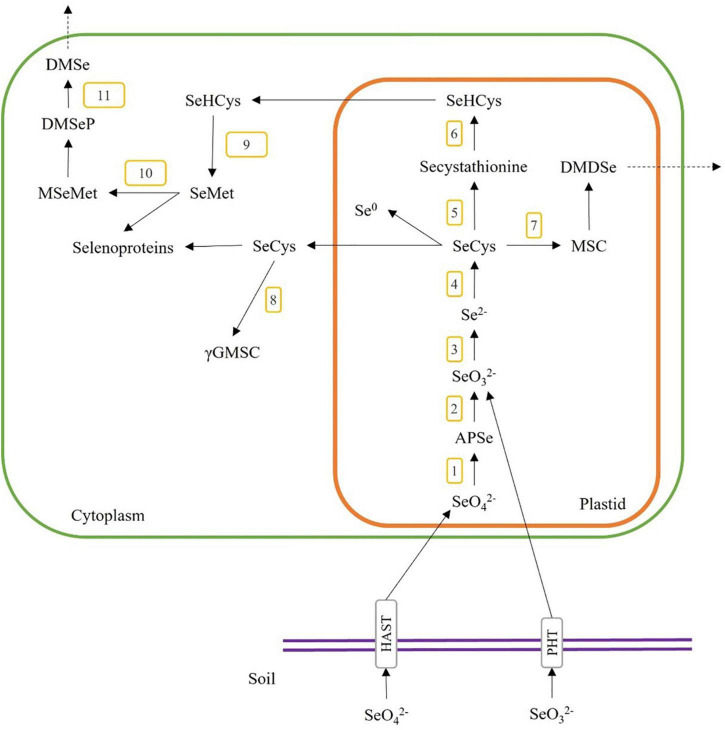
A general overview of Se uptake, metabolism, and incorporation in higher plants. The numbers 1–12 indicate the possible enzymatic steps involved in the conversion of selenite and selenate. 1, ATP sulfurylase; 2, adenosine phosphosulfate reductase; 3, sulfite reductase; 4, O-acetylserine thiol-lyase; 5, cystathionine-γ-synthase; 6, cystathionine-β-lyase; 7, selenocysteine-lyase; 8, γ-glutamyl-cysteine synthetase; 9, methionine synthase; 10, methionine methyltransferase; 11, dimethyl selenoproprionate-lyase; SeO${}_{4}^{2-}$, selenate; SeO${}_{3}^{2-}$, selenite; APSe, adenosine 5′-phosphoselenate; SeCys, selenocysteine; MSC, Se-methylselenocysteine; DMDSe, dimethyldiselenide; SeHCys, selenohomocysteine; Se^0^, elemental selenium; γGMSC, γ-glutamyl-methylselenocysteine; SeMet, selenomethionine; MSeMet, methyl-selenomethionine; DMSeP, dimethylselenoproprionate; DMSe, dimethyl selenide.

As the Se content and chemical form in plant materials are specific to the plant species and their metabolism pathways, we need to understand the Se accumulation mechanisms in the plant when selecting plant species for producing Se-enriched plant foods and food ingredients for human diets. The accumulation pathways of Se content start with the inorganic Se (i.e., selenite and selenate) in soil, which plants could uptake and transform into organic forms (i.e., selenocystine (SeCys_2_), selenomethionine (SeMet), selenohomocysteine, selenolanthionine Se-methylselenocysteine (MSC) and γ-glutamyl-methylselenocysteine (γGMSC)) through the metabolic pathways as shown in Figure 2. Briefly, selenate and selenite are taken through the plant root via high-affinity sulphate transporter (HAST) and high-affinity phosphate transporter (PHT). Selenate is converted to adenosine 5’-phosphoselenate (APSe) via ATP sulfurylase (Figure 2, step 1), then changed to selenite through adenosine phosphosulfate reductase (Figure 2, step 2). Selenite is reduced to selenide (Se^2–^) by sulphite reductase (Figure 2, step 3), and then it is transformed to selenocysteine (SeCys) by O-acetylserine thiol-lyase (Figure 2, step 4). SeCys could also be transformed to Se-cystathionine via cysthathionine-γ-synthase (Figure 2, step 5), MSC via selenocysteine-lyase (Figure 2, step 7), or elemental selenium (Se0). Secystathionine could then be changed into selenohomocysteine (SeHCys) via cysthathionine-β-lyase (Figure 2, step 6). MSC could be converted to dimethyldiselenide (DMDSe), a volatile compound and released from plant cells. SeCys is transported to the cytoplasm and is reacted with glutamic acid to form γ-glutamyl-Se-methylselenocysteine (γGMSC) by γ-glutamyl-cysteine synthetase (Figure 2, step 8). SeHCys can also be transported to the cytoplasm and synthesized to form selenomethionine (SeMet) by methionine synthase (Figure 2, step 9). SeMet could also be converted to methyl-selenomethionine (MSeMet) by methionine methyltransferase (Figure 2, step 10), then changed to the volatile dimethylselenoproprionate (DMSeP) and released as dimethyl-selenide (DMSe) via dimethylselenoproprionate-lyase (Figure 2, step 11) (27–29).

During the accumulation process, selenite tends to provide higher bioavailability than selenate, and it is commonly used as Se fertilizer for producing Se enriched plants (30, 31). Hu et al. (24) showed that using selenite as the foliar fertilizer on rice grain increased the Se concentrations in glutelin and albumin proteins as SeCys_2_ and SeMet. Selenite could cause significant phytotoxicity from a generation of superoxide in plant cells during a non-enzymatic reduction reaction to produce selenide (25, 32, 33). In another study, Ramkissoon et al. (34) applied sodium selenate to wheat as foliar fertilizer and found an increased Se concentration and the highly bioavailable SeMet fraction in wheat grain. However, Se can cause cytotoxicity in plants and humans when accumulated or consumed excessively. At high concentrations, Se shows cytotoxicity by either generating reactive oxygen species or malformed selenoprotein (20). Generally, inorganic Se, either selenite or selenate, generates toxicity via the activation of ROS, which inhibits the growth rate and causes lipid oxidation related to malondialdehyde formation in plant tissue (35, 36).

In contrast, organic Se, such as SeMet and SeCys, cause toxicity to plant cells by forming malformed selenoproteins due to the replacement of Cys/Met with SeCys and SeMet in the peptide chain. Changing between Cys and SeCys changes cellular protein’s structure by changing disulfide bond to diselenide bond to 60 mg Se kg^–1^, which affects the peptide chain’s redox potential. Moreover, SeCys is more reactive than Cys, which could increase enzyme activity and the metal binding co-factor activity of malform selenoproteins (27). Literature has shown that organic Se’s toxicity level is far less than inorganic ones because they can be capped with proteins and polysaccharides (37). Moreover, the organic Se compounds display a higher bioavailability than the inorganic Se (38). The organic Se involves in the upregulation of enzymatic antioxidant capacity which play a key role in Se tolerance (39). As the Se chemical forms significantly affect the biological activities of Se-enriched plants, it is essential to perform chemical speciation of Se compounds to gain scientific insight into the relationship between chemical forms and the functional properties of Se-enriched plant foods.

## Speciation of selenium compounds

Speciation of Se compounds in Se-enriched plant foods has been studied to relate to and explain the biological activity of the products. Se can accumulate in plant organelle, stay either in free molecules form, or bound with a larger and more complex structure such as polypeptides or polysaccharides. Most inorganic Se compounds and small selenoamino acids such as selenolanthionine, γGMSC, MSC, SeCys_2_ and SeMet are water-soluble molecules, therefore water extraction is a common method applied to separate these small molecules from the sources. Proven in some previous studies, extraction efficiencies in hot water ranged between 47 and 91% Se in different mushroom species (40); 40% for Se-enriched mycelium (*Lentinula edodes* (Berk.) *Pegl.*) (41), 85% for Se-enriched garlic (42) and 60% for *Cardamine violifolia* (26). Multiple sample preparation steps have been used to release Se bind to some larger components in plant cell walls. For example, hydrolysis of polysaccharides using an enzyme such as cellulase, hemicellulose, β-glucanase and pectinase, has been applied to hydrolyze plant cell walls, followed by protease enzymes to release selenoamino acids (43, 44).

Selenium compounds extracted from the plants could be separated by the High-Performance Liquid Chromatography (HPLC) technique, commonly used in the chemical compound analysis. Various types of chromatography resin can be used to separate the specific Se compounds in plants. For example, ion-exchange chromatography is used in the scouting period, which can classify Se chemical compounds according to their electron charge binding to ion exchange resins, either in a negatively charged resin (cation exchanger) or a positively charged resin (anion exchanger) (45, 46). Thus, ion-exchange chromatography is the technique that separates Se molecules by the positively or negatively charged groups retained on a stationary phase in equilibrium with free counter ions in the mobile phase (47). Generally, when the pH of the eluent buffer is higher than the pKa of the molecule, the compound shows a negative charge and binds to the positive charge anion exchanger (46, 48). Anion exchange liquid chromatography has a positively charged stationary phase to interact with the negatively charged Se compounds, such as selenate (p*K*a = 1.92), selenite (p*K*a = 2.46) or SeMet (p*K*a_1_ = 2.19 and p*K*a_2_ = 9.05) in the deprotonated state which can be strongly retained on anion exchange resin at pH around 5. In contrast, Se compounds with higher p*K*a values, such as SeCys_2_ (p*K*a ∼ 8.07 and 8.94), will be in protonating state and retained very little on anion column chromatography at pH around 5 in the mobile phase (49, 50). In contrast, cation exchange chromatography works similarly to anion exchange, except that the stationary phase is negatively charged, which could interact with the positively charged Se compounds (51, 52). Furthermore, some other types of chromatography could be applied for Se compound separation. For example, size exclusion chromatography is used to separate compounds based on their particle size; reversed phase and hydrophilic interaction chromatography could be applied to separate Se compounds based on the polarity of their molecules (51). These types of chromatography can be applied simultaneously to identify different Se compounds in plant extracts.

After the chromatographic separation, the mass of Se molecules can be detected by techniques such as the Inductively Coupled Plasma-Mass Spectrometry (ICP-MS) or Inductively Coupled Plasma-Optical Emission Spectrometry (ICP-OES). These techniques detect Se molecules based on their transition ions which provide high accuracy detection, low detection limit (part per trillion), and less matrix interference (53, 54). The HPLC-ICP-MS has been considered a robust workflow and is widely used for Se determination in Se-containing plants and foods. A study by Ogra et al. (55) successfully applied size-excursion chromatography incorporated with ICP-MS to identify the Se metabolic pathway of ginger and Indian mustard using selenate or SeMet as Se fertilizers. The study found that γ-Glutamyl-Se-methylselenocysteine and MSC were the common metabolites of selenate and SeMet in garlic and Indian mustard.

As mentioned earlier, the Se compounds accumulated and stored differ by plant genus/species, and some Se can be bound to highly complex structure. In addition to the methods described above, other technique can be applied to identify the Se compounds started with compound purification by ion-exchange chromatography, followed by identification of the molecular mass by Electrospray Ionization-Mass Spectrometry (ESI-MS) (26, 56, 57). The ESI-MS is a technique that ionizes chemical compounds by electrospray ionization, and a mass analyzer then detects the ionized molecules according to their mass/charge (m/z) ratio (58). This high sensitivity mass spectroscopy technique can provide effective approaches to the speciation of Se bound in complex structures such as selenosugars and selenoproteins (59, 60). Some novel analysis methods have also been used to specify Se compounds in food materials. For example, Laser Ablation-Inductively Coupled Plasma-Mass Spectrometry (LA-ICP-MS) is a solvent free analytical technique used to analyze Se compound in solid sample and it can provide greater accuracy results compared to traditional liquid chromatography (61). Moreover, the X-Ray Absorption Spectroscopy (XAS) technique was used to identify Se compounds in biological sample with less sample preparation step to prevent the degradation of Se compound from chemical reaction during sample preparation (62). These analytical techniques can be valuable to identify any specific and new-found Se compounds in plants that could then be studied to understand their biological activity in the Se-enriched plant food products.

## Bioactivity of Se compounds

Generally, literature shows that organic Se species tend to have higher bioactivities, bio-accessibility and lower toxicity than inorganic Se species. Research in human immortalized keratinocytes (HaCaT) cells showed that SeMet had a lower cytotoxicity effect on HaCaT cells than sodium selenite, where the IC_50_ of SeMet was 55.4 μM, much higher than 2.3 μM from sodium selenite (63). The lower cytotoxicity might be related to the antioxidant activity of organic Se compounds to prevent toxicity and cellular damage by increasing selenoamino acid and selenoproteins, which could enhance the activity of antioxidant enzymes such as glutathione peroxidase and thyroxine reductase (19, 64). For example, SeMet had increased GPx activity in rat skin cells at a higher dose than inorganic Se (selenite), which caused a toxic effect at 1μM (Hazane-Puch et al. (63). Moreover, SeMet increased the GPx activity and total antioxidant content while lower MDA formation in broiler chicken tissue compared to the sodium selenite-treated subjects (65).

**FIGURE 3:**
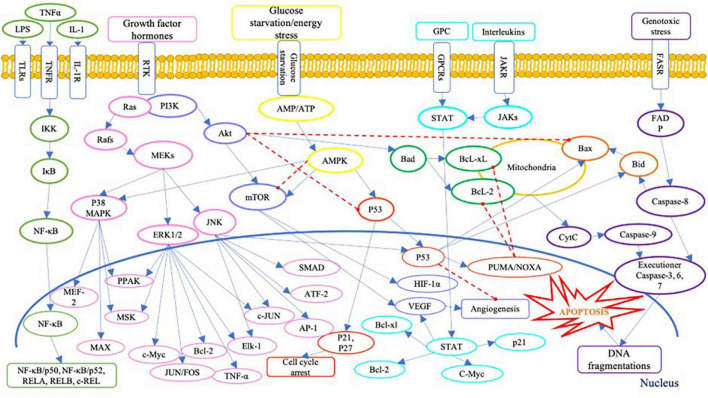
A schematic of apoptosis signaling pathways. LPS, lipopolysaccharide; TNFα, tumour necrosis factor alpha; IL-1, interleukin-1; TLRs, Toll-like receptors; TNFR, tumor necrosis factor receptors; IL-1R, interleukin-1 receptor; GPC, G protein complex; GPCRs, G protein-coupled receptor; JAKR, Janus kinase receptor; FASR, Fas receptor; IKK, IκB kinase; IκB, inhibitor of NF-κB; NF-κB, nuclear factor (NF)-κB; REL, REL protein; Ras, Ras protein; Rafs, Raf kinases; MAPK, mitogen-activated protein kinase; MEKs, MAPK/ERK kinase; ERK, extracellular signal-regulated kinase; JNK, c-Jun N-terminal kinases; MEF-2, myocyte enhancer factor-2; PPAK, family of p21-activated protein kinases; MSK, mitogen and stress activated protein kinase; MEK, mitogen-activated protein kinase; MAX, MAX protein; c-Myc, c-Myc protein; JUNFOS, Fos and Jun families of DNA binding proteins; Bcl-2, B-cell lymphoma 2; ELK-1, ETS transcription factor ELK1; AP-1, activator protein 1; ATF-2, activating transcription factor 2; PI3K, phosphoinositide 3-kinases; Akt, serine/threonine-protein kinases; mTOR, mammalian target of rapamycin; HIF-1α, hypoxia inducible factor 1 subunit alpha; VEGF, vascular endothelial growth factor; AMP, adenosine monophosphate activated protein; ATP, adenosine triphosphate; AMPK, AMP-activated protein kinase; p53, protein p53; PUMA, p53 upregulated modulator of apoptosis; NOXA, (PMAP1) – phorbol-12-myristate-13-acetate-induced protein 1; Bcl-xl, B-cell lymphoma-extra large; Bad, Bcl-2 associated death promoter; Bax, Bcl-2 associated protein x; Bid, BH3 interacting domain death agonist; STAT, signal transducer and activator of transcription; JAKs, Janus kinases; FADP, flavin adenine dinucleotide; cytc, cytochrome complex (187–192).

On the other hand, the presence of Se compound in high concentration could generate cytotoxicity in human cells. Literature has identified several cytotoxic pathways of Se compounds across various human cancer cell lines (Table 1). Inorganic Se species, i.e., sodium selenite, was widely studied, especially on prostate cancer cells. The cellular toxicity mechanism of sodium selenite against human prostate cancer cells has been identified as below: generation of anti-proliferation effect via the expression of mRNA of the SELV, SELW, and TGR selenoproteins (66); promotion of GLS1 protein degradation and APC/C-CDH1 apoptosis pathways (67); induction of cell apoptosis via activation of caspase-8 protein (68); and activation of p53 protein (69). Moreover, the anti-proliferation activity of inorganic Se, including sodium selenite, has been reported in human lung cancer cell lines; it has involved inhibiting the Trx1 expression (70). Several signaling pathways are involved in cell anti-proliferation and apoptosis in human cells, as shown in Figure 3. Briefly, Se could cause cell death via apoptosis pathways by activating the executioner caspase-3, 6, 7, and 9, and promoting pro-apoptosis genes Bax and Bid on mitochondria and producing cytochrome C (CytC). The toxic effect of Se compounds could also mediate DNA repair and cell angiogenesis by promoting pro-apoptosis genes, including Bax and Bid (71).

**TABLE 1:** Cytotoxicity of Se compounds against human cancer cell lines.

Tumor organs	Cell lines	Se compounds	Effective doses (IC_50_)	Cell viability method	Mechanism of cell death	References
Lung	A549	SeMet	50 μM	MTT Assay	• Induce ROS generation • Induce ER stress-related to p53 regulation	(73)
		SeMet	50 μM	MTT Assay	• Induce ROS generation Interrupt PI3K/Akt/mTOR pathway	(117)
		SeMet	500 μM	MTT Assay	• Induce ROS generation	(74)
		SeMet	200 μM	Cell counting kit-8	• Induce ROS generation • Increase the intensity of the mitochondrial membrane	(118)
		MSC	50 μM	MTT Assay	• Activate caspase-3,-8,-9 • Interrupt PI3K/Akt pathway • Induce ER stress	(73)
		SeCys_2_	5 μM	ATP measurement	• Induce ROS generation • Decrease total cellular glutathione	(119)
		SeCys_2_	8 μM	MTT Assay	• Induce ROS generation • Induce loss of mitochondria membrane by regulating Bcl-2 family proteins • Induce apoptosis via inactivating ERK and AKT pathways	(77)
		MSA	2.2 μM	MTT Assay	• Induce DNA single strand break • Induce apoptosis via cell cycle arrest G1 phase	(120)
		Nano-Se	4 μM	MTT Assay	• Induce apoptosis via cell cycle arrest G2/M phase	(121)
	95-D	MSA	4 μM	MTT Assay	• Induce ROS generation and oxidative damages	(122)
Breast	MCF-7	SeCys_2_	10 μM		• Induce H_2_O_2_ production • Decrease mitochondria protein UCP2 and MnSOD	(123)
		SeCys_2_	16.2 μM	MTT Assay	• Induce DNA single strand break • Induce ROS generation • Decrease cellular antioxidant enzymes	(124)
		MSA	2 μM	FACS CANTO II	• Induce apoptosis via cell cycle arrest G2/M phase • Inhibit DNA methyltransferase 1 (DNMT1)	(125)
	MCF-7	SeMet	45 μM	SRB Assay		(126)
		SeCys_2_	40.8 μM	CCK-8 assay	• Induce apoptosis via cell cycle arrest G1 phase	(127)
Colon	HCA-7	SeMet	60 μM	SRB Assay	• Inhibit cyclooxygenases-2 (COX-2) protein	(128)
	HT-29	SeMet	130 μM	SRB Assay		(126)
	HCT116	SeMet	100 μM	Propidium iodide staining	• Induce apoptosis via cell cycle arrest G2/M phase • Decrease mitotic cyclin B RNA expression • Decrease cdc2 kinase activity	(129)
	SW620	SeCys_2_	7.3 μM	MTT Assay	• Induce DNA single-strand break • Induce ROS generation • Decrease cellular antioxidant enzymes	(124)
	Colo201	SeCys_2_	27.8 μM	MTT Assay	• Induce DNA single-strand break • Induce ROS generation • Decrease cellular antioxidant enzymes	(124)
Prostate	LNCaP	SeMet	50 μM	Model Z F Coulter Counter	• Increase p53 gene expression	(130)
		SeMet	1 μM	Growth Inhibition Assay	• Induce apoptosis via cell cycle arrest G2/M phase	(131)
	DU145	SeMet	40 μM	SRB Assay	–	(126)
		SeMet	90 μM	Growth Inhibition Assay	• Induce apoptosis via cell cycle arrest G2/M phase	(131)
		MSA	5 μM	MTT Assay	• Induce apoptosis via inactivation of protein kinase C (PKC)	(132)
	PC-3	SeMet	70 μM	Growth Inhibition Assay	• Induce apoptosis via cell cycle arrest G2/M phase	(131)
Liver	HepG2	SeCys_2_	17.5 μM	MTT Assay	• Induce DNA single-strand break • Induce ROS generation • Decrease cellular antioxidant enzymes	(124)
		Selenosulfate	>15 μM	MTTAssay	–	(133)
Bone	MG-63	SeCys_2_	20 μM	MTT Assay	• Induce apoptosis via cell cycle arrest G2/M phase • Decrease cyclin A and CDK-2, PARP cleavage, and caspases activation	(134)
Urinary bladder	T24	Selenosulfate	3.5 μM	MTT Assay	–	(133)
Brain	IPSB-18	Sodium selenite	4 μg/ml	MTT/SRB Assay	• Downregulation metalloproteases genes and epidermal growth factor receptor	(135)
Oral	HSC-3	MSC	>50 μM	MTTAssay	• Enhance activity of caspase-3, -8, -9 • Induce ER stress • Reduce phosphorylated Akt levels and vascular endothelial growth factor (VEGF).	(73)
Skin	UACC-375	SeMet	50 μM	SRB Assay	–	(126)
	A375	SeCys_2_	12.8 μM	MTT Assay	• Induce DNA single strand break • Activate caspase peptides • Induce p53 expression	(136)
		Selenosulfate	4.7 μM	MTT Assay	–	(133)
		SeCys_2_	20 μM	MTT Assay	• Upregulate genes encoding cell death and transcription factors • Downregulate cell development, cell adhesion and cytoskeleton genes	(137)
Cervix	HeLa	SeCys_2_	99.5 μM	XTT cell proliferation kit II	• Upregulate apoptosis gene BCL2L11 and DNA damage GADD45G • Induce cytoplasmic vacillation via LC-3II protein formation • Induce ER stress by decreasing ER-residing protein	(138)

SeMet, Selenomethionine; MSC, Se-methylselenocysteine; SeCys_2_, selenocystine; MSA, methylseleninic acid; ROS, reactive oxygen species.

A high concentration of Se compounds also performs a redox-active act as prooxidants, generating ROS in reaction (72). The redox action of Se compounds that generate ROS in the human cell could be the primary focus when using Se as an anticancer agent against human cancer cells. According to some studies (Table 1), SeMet could inhibit cell proliferation by inducing ROS generation and activating apoptosis cellular proteins, including the caspase family and p53 (73, 74). The ability to generate ROS could meditate the toxicity of Se due to the production of oxidative stress involved in cell cytotoxicity and apoptosis induction (75, 76). Moreover, MSC can induce cancer cell apoptosis via an interface of cell proliferation PI3K/Akt pathway (73), while SeCys_2_ downregulated Bcl-2 survival genes in lung cancer cell lines (77). A study by Hui et al. (78) also showed that selenite induced cell apoptosis by upregulate cell death protein p38 MAPK and inhibition of the PKD1/CREB/Bcl-2 survival pathway.

The current research on Se compounds focuses on both sides of the spectrum: the protective effect against cell damage or the anti-proliferation effect against cancer cell lines. Se compounds’ bioactive information could impact the functional properties of Se-enriched plant foods, not only the concentration of Se in the sample but also the chemical form of Se accumulated. Besides, the bioactive compounds such as polyphenol, polypeptides and polysaccharides in plant foods could also significantly affect the uniqueness of bioactivities and functional properties of Se enriched plants.

## Biological properties of Se-enriched plant foods

**FIGURE 4:**
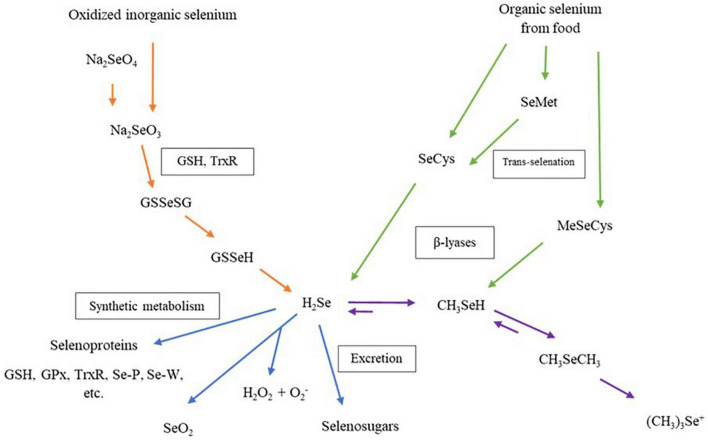
Metabolism of dietary selenium compounds in human cells. Na_2_SeO_4_, sodium selenate; Na_2_SeO_3_, sodium selenite; GSH, glutathione; TrxR, thioredoxin reductase; GSSeGS, selenodiglutathione; GSSeH, glutathioselenol; H_2_Se, hydrogen selenide; GPx, glutathione peroxidase family; Se-P, selenoprotein P; Se-W, selenoprotein W; SeO_2_, selenium dioxide; H_2_O_2_, hydrogen peroxide; SeCys, selenocysteine; SeMet, selenomethionine; MeSeCys, methylselenocysteine; CH3SeH, selenol; CH_3_SeCH_3_, dimethylselenide, (CH_3_)_3_Se^+^, trimethylselenonium ion.

The biological properties of Se-enriched plant foods have received more interest from researchers in the past two decades. Figure 4 shows that the Se compound in Se-enriched plant foods induces biological activities through different metabolism pathways in human cells. Metabolism pathways of Se compounds begin with a reduction of inorganic or organic Se compounds from food supplements to hydrogen selenide (H_2_Se). This H_2_Se will be metabolized and synthesized into several selenoproteins, then transported and stored in human organs (79, 80). More than 25 selenoproteins have been identified in human cells, and some are considered antioxidant enzymes, such as glutathione peroxidase (GPxs), iodothyronine deiodinases, thioredoxin reductases (TrxR). These individual selenoproteins perform biological properties, including balancing plasma glucose levels and insulin sensitivities, anti-inflammatory and enhancing cell proliferation (4).

At their non-toxic concentration, Se-enriched plants could protect against cellular damage from hydrogen peroxide (H_2_O_2_) stress and enhance antioxidant enzymes in normal human cells. Table 2 shows a compilation of research on the health effects of Se-enriched plants using *in vitro* human cells models. The antioxidant effect of Se-enriched food products has prevented oxidative stress induced by H_2_O_2_ in human cell lines. For example, Se-enriched polysaccharides extracted from *Pleurotus ostreatus* and Se-enriched rice grass extract showed a protective effect against cellular oxidative stress from H_2_O_2_-induction in human muscle and human kidney cells (81, 82). Moreover, Se-enriched soybean peptide increased the activities of cellular antioxidant enzymes, including GPx, SOD, and CAT, in human colon cells (83, 84).

**TABLE 2:** *In vitro* studies of Se-enriched plant foods against human cell lines.

Se-enriched food	Cell lines	Concentrations	Mechanisms/Pathways	References
Kale and kohlrabi sprouts	Human colon cancer cells (SW480, SW620), liver cancer cell (HepG2), uterus (SiHa) cells	1 mg ml^–1^	Inhibit cell growth	(139)
Konjac glucomannan	Human lung cancer (A549), breast cancer (HCC1937) cells	0.15-0.6 μg ml^–1^	↑Mitochondria apoptosis ↑Cleaved caspase-3 and ↑PARP-activated fragments	(140)
Polysaccharide from *Malus toringoides* (Rehd.) Hughes	Human liver cancer (HepG2) cells	50-200 μg ml^–1^	↓ROS generation ↓H_2_O_2_ induction	(141)
Polysaccharide from alfalfa root	Human liver cancer (HepG2) cells	140 μg ml^–1^	Inhibit cell growth	(142)
Soybean peptide	Human colon tumor cells (Caco-2)	10 μg ml^–1^	↓ H_2_O_2_ induction ↑GPx; ↑SOD; and ↑CAT	(84)
Polysaccharide from *Tithonia* *diversifolia* (Hemsley) A.	Human gastric cancer (MKN7) cells	72.9-92.6 μg ml^–1^	Inhibit cell growth	(143)
Hawthorn fruit	Human liver cancer (HepG2) cells	19.2 μg ml^–1^	↑ROS generation ↑Caspase-9 ↓Blc-2	(86)
Broccoli sprout	Human prostate cancer (LNCaP) cells	0.27 μg ml^–1^	↓PI3K/Akt/mTOR pathway	(144)
Polysaccharide from *Pleurotus ostreatus*	Human murine skeletal muscle (C_2_C_12_) cells	400 μg ml^–1^	↓H_2_O_2_ generation Inhibit cell apoptosis	(82)
Ricegrass	Human kidney Cell (HEK293) cells	10 mg ml^–1^	↓MDA ↓Oxidative stress and DNA damage	(81)
Kale roots	Human liver cancer (HepG2) cells	20 mg ml^–1^	↑Nrf_2_ protein	(145)
Se-enriched *Astragalus polysaccharide*	Human liver cancer (HepG2) cells	10 mg ml^–1^	Inhibit cell growth	(146)
Polysaccharide from *Cordyceps gunnii*	Human ovarian cancer (SKOV3) cells	0.4 mg ml^–1^	↑Cleavage caspase-3, -9, ↑PARP and ↑BAX ↓Bcl-2	(147)
Polysaccharide from *Rosa laevigata*	Human neuroblastoma (SH-SY5Y) cells	0.1 mg ml^–1^	↓H_2_O_2_ generation	(148)
Polysaccharide from *Ginkgo biloba* L. leaves	Human bladder cancer (T24) cells	200 μg ml^–1^	↑Cleavage caspase-3, -9, ↑PARP and ↑BAX ↓Bcl-2	(149)
Polysaccharide from *Pyracantha fortuneana*	Human breast cancer (MDA-MB-231) cells	400 μg ml^–1^	Inhibit cell growth via cycle arrest at G2-phase ↑p53; ↑Bax; ↑Puma; ↑Noxa ↑Casepase-3,-9.↓Bcl2	(150)
Polysaccharide from *Pyracantha fortuneana*	Human ovarian cancer (SKOV3, HEY) cells	400 μg ml^–1^	↑PARP; ↑Cleavage caspase-3; ↑Bax; ↓Bcl-2	(94)
Broccoli seed	Human Glioblastoma astrocytoma (U215) cells	28.5 μg ml^–1^	Inhibit cell growth	(151)
Cauliflower	Human colon tumor (Caco-2) cells	2,500 μg ml^–1^	Inhibit cell growth Changing cell morphology	(152)
Ziyang green tea	Human breast cancer (MCF-7) cells	172.2 μg ml^–1^	Cycle arrest at G0/G1-phase ↑p53; ↑ Bax/Bcl-2 ratio; ↑caspase-3, -9; ↑ROS	(153)

↑, increase or upregulate; ↓, decrease or downregulate; Akt, protein kinase B; BAX, B-cell lymphoma 2 associated X; Blc-2, B-cell lymphoma 2; CAT, catalase; GPx, glutathione peroxidase; H_2_O_2_, hydrogen peroxide; MDA, malondialdehyde; mTOR, mammalian target of rapamycin; NOXA, phorbol-12-myristate-13-acetate-induced protein 1; Nrf2, nuclear factor erythroid 2–related factor 2; PARP, Poly (ADP-ribose) polymerase; PI3K, phosphoinositide 3-kinase; PUMA, p53 upregulated modulator of apoptosis; p53, tumor protein 53; ROS, reactive oxygen species; SOD; superoxide dismutase.

In contrast, Se-enriched plants could generate cellular ROS and influence cell death via the apoptosis mechanism at their toxic concentrations. For example, with human cancer cell lines, Se-konjac glucomannan performed anti-proliferation properties against human lung cancer cells (A549) and human breast cancer cells (HCC1937) by activating mitochondria pro-apoptosis protein caspase-3 (85). Furthermore, Se-enriched hawthorn fruit induced cellular apoptosis on human liver cancer (HepG2) cells by upregulation of pro-apoptosis protein caspase-9, downregulation of anti-apoptosis protein Blc-2, and increasing intracellular ROS level (86). These findings indicated that Se-enriched plant foods could perform both proliferation and anti-proliferation on either cancer or non-cancer cell lines and the effects depend on Se’s dose and chemical forms in the diets.

Table 3 shows positive results on the biological properties of Se-enriched plants and some food ingredients (microalgae, probiotics bacteria and milk casein) in the *in vivo* animal models compared with Se-enriched yeast, an alternative source of SeMet (around 60-84%) with a lower toxic effect (87, 88). Various bioactive effects have been reported from Se-enriched plants, including increasing Se content in animal serum and tissue, enhancing antioxidant enzymes, lowering lipid oxidation in liver-stress animals, upregulation of cellular proliferation proteins, and downregulation of pro-inflammation and apoptosis cellular proteins. Some food products, for example, Se-enriched *Auricularia auricular* mushroom and Se-enriched radish sprouts, showed similar effects on improving antioxidant activities such as GPx and catalase, lower malondialdehyde (MDA) levels, and protecting liver damages in high-fat diet mice (89, 90). Se-polysaccharide from *Astragalus* also has anti-inflammatory effects on diabetic mice by lower serum inflammation-related proteins, including C-reactive protein, tumor necrosis factor-alpha (TNF-α), interleukin-6 (IL-6) and nuclear factor kappa B (NFκB) (91, 92). Moreover, Se-polysaccharide purified from *Pyracantha fortuneana*, and Se-enriched sweet potato inhibited tumor growth via apoptosis pathway and decreased IL-2, TNF-α, and VEGF in mice xenograft with human cancer tumor (93, 94).

**TABLE 3:** *In vivo* studies of Se-enriched plants and other food materials using animal models.

Se-enriched food or materials	Animal models	Treatment	Functional properties	References
Olive leaves	Growing rabbits	Treated with 2.17 mg Se kg^–1^ per dry leaves extract for 70 days	↑Serum antioxidant ↓Leukocyte DNA damage	(154)
Radish sprouts	CCl_4_-induced liver injury mice	Treated with Se-enriched radish sprout in combination with inorganic Se compounds for 6 weeks	↓Inflammatory reaction in liver tissue ↓MDA in liver tissue ↑GPx in liver tissue	(90)
Gallic and cabbage	Broilers	Fed with a mixture of Se-gallic and cabbages	↑Se content in plasma ↑GPx in plasma	(155)
Garlic polysaccharide	Mice	Injected with 0.6 mg Se-polysaccharide	↑TNF-α; ↑IL-6; ↑IL-1 in macrophages	(156)
Radish sprout	Tumorigenesis induced rats	Treated with 12.5 ppm per day for 3 weeks	↑GPx; ↑GST in liver and lung	(157)
Kale spout	Male broilers	Treated with 2 mg Se kg^–1^ per day 42 days	↑Se content in animal tissue ↑GPx in plasma	(158)
Lotus leaf polysaccharide	Gestational Diabetes rats	Treated with 100 mg kg^–1^ per day for 7 weeks	↑GSH content, ↑GPx; ↑SOD; ↑CAT ↓FBG, ↓TG, triglyceride, LDL content.	(159)
Ziyang green tea polysaccharide	Chronic fatigue syndrome rats	Treated with 200 mg kg^–1^ per day for 4 weeks	↑Corticosterone ↓Aldosterone serum hormones	(160)
Rice	Diabetic mice	Treated with 0.2 mg g^–1^ body weight of 250 g L^–1^ Se-rice for 4 weeks	↓ C-reactive protein; ↓TNF-α; ↓IL-6; ↓COX-2 and ↑NFκB in serum	(91)
Wheat	Broilers	Treated with 37-185 μg Se kg^–1^ per day for 21 days	↑Se content in muscle and liver	(161)
Soybean peptide	Male Kunming mice	Treated at 4 mg Se kg^–1^ per day for 7 days	↑SOD in liver tissue ↓MDA in liver tissue	(84)
Soybeans	CCl_4_-induced liver injury rats	Treated with 700 mg kg^–1^ twice a week for 8 weeks	↓α-SMA in the liver ↑mRNA expression of MMP9 ↑GSH; ↑GPx in liver tissue	(162)
Yellow pea and oat polysaccharides	Male weanling Sprague-Dawley rats	Treated with 40 μg Se kg^–1^ per day for 50 days	↑GPx in blood and liver ↑TrxR1 in liver	(163)
Soy protein isolate	Male weanling Sprague–Dawley rats	Treated with 30 μg Se kg^–1^ per day for 50 days	↑GPx in blood and liver ↑TrxR1 in liver	(164)
*Auricularia auricular* mushroom	High-Fat Diet Streptozotocin-induced diabetic mice	Treated at 500-1,000 mg kg^–1^ for 8 weeks	↓Diabetes-induced disorders of lipid metabolisms; ↓Liver damage ↑GPx; ↑CAT; ↓MDA in liver tissue	(89)
*Grifola frondosa* mushroom polysaccharide	Cyclophosphamide induced mice	Treated with 120 mg kg^–1^ per day for 7 days	↑GPx; ↑SOD; ↑CAT in serum, liver and kidney	(165)
*Astragalus* mushroom polysaccharide	CCl_4_-induced liver injury rats	Treated with 40 mg per day for 7 weeks	↓TNF-α; ↓IL-6; ↓COX-2; ↓NFκB in liver tissue ↑Bcl-2/Bax ratio in liver tissue	(92)
Sweet potato polysaccharide	Hepatoma (H22) cells xenograft mice	Injected with 100 mg kg^–1^	↑IL-2; ↑TNF-α; ↑VEGF in serum ↓Tumor growth ∼58%	(93)
*Hypsizigus marmoreus* polysaccharide	CCl_4_-induced liver injury mice	Treated with 800 mg kg^–1^ per day for 10 days	↓MDA; ↓Lipid oxidation in serum and liver ↑GPx; ↑SOD in serum and liver	(166)
*Pyracantha fortuneana* polysaccharide	Human ovarian carcinoma (HEY) cells xenograft mice	Treated with 400 mg Se day^–1^ for 28 days	↓Cancer cell proliferation; ↑apoptosis ↓Cytoplasmic β-catenin	(94)
*Pyracantha fortuneana* polysaccharide	CCl_4_-induced liver injury Kunming mice	Treated with 400 mg kg^–1^ per day for 5 weeks	↑GPx; ↑CAT in liver ↓TBAR; ↓ H2O2 in liver	(167)
*Catathelasma ventricosum mycelia.*	Streptozocin-induced diabetic mice	Treated with 500 mg kg^–1^ per day for 30 days	↑GPx; ↑SOD; ↑CAT; ↓ MDA in liver tissue	(168)
*Agaricus bisporus* mushroom	Hyperthermal induced oxidative stress rats	Treated with 1 μg Se g^–1^ per day for 5 weeks	↑GPx in *ex vivo* ileum	(169)
*Pleurotus ostreatus* mushrooms	Wistar male rats	Treated with 0.15 mg Se kg^–1^ per day for 21 days	↑Se content in plasma	(170)
*Microalgae*	Yearling common barbel fishes	Treated with 1 mg Se kg^–1^ per day for 6 weeks	↑GR in muscle and liver ↑Alanine aminotransferase; ↑Creatine kinase in blood plasma	(171)
*Candida utilis*	Sprague-Dawley rats	Treated with 3 mg Se kg^–1^ per day for 6 weeks	↑GPx; ↑SOD; ↑CAT; ↑GSH in serum and liver	(172)
*Lactobacillus acidophilus*	High-fat diet mice	Treated with 0.3 μg Se per day for 4 weeks	↑GPx; ↑SOD in serum ↓MDA; ↓TC; ↓TG; ↓LDL in serum	(173)
*Lactobacillus acidophilus* and Se-yeast	Crossbred weanling piglets	Treated with 0.46 mg Se kg^–1^ per day for 42 days	↑GPx in blood ↑TrxR mRNA in tissue	(174)
*Lactobacillus acidophilus* and Se-yeast	CCl_4_-induced liver injury rats	Treated with 0.05 mg kg^–1^ Se per day for 7 weeks	↑GPx; ↑GSH; ↑SOD; ↓ MDA in liver tissue ↓ TNF-α; ↓IL-6; ↓MCP-1 in liver tissue	(175)
Milk casein isolate	Human epithelial breast cancer (MCF-7) cells xenograft mice	Treated with 1.15 μg Se g^–1^ per day for 70 days	↓Tumor volume ↑Apoptotic cells	(176)
Se-milk protein and yeast	Mice	Treated with 1 μg Se g^–1^ per day of either Se-milk protein or Se-yeast for 4 weeks	↑selenoprotein P; ↑GPx-2 in colon Only Se-yeast ↑GPx1	(177)
Se-yeast	Hepatotoxicity chickens	Treated at 50 μg kg^–1^ per day for 21 days	↓ALT; ↓AST; ↓MDA in serum ↑GPx; ↑SOD in serum	(178)
Se-yeast	Ochratoxin A-induced small intestinal injury chickens	Treated at 0.4 mg kg^–1^ per day for 21 days	↓Intestinal injury from ochratoxin A-induction via Nrf2 pathway ↓NF-κB activation	(179)
Se-yeast	5-fluorouracil induced mice	Treated with Se-yeast at 10^8^ CFU per day	↓Eosinophil peroxidase activity; ↓CXCL1 levels; ↓Histopathological tissue damage ↓Oxidative stress.	(180)
Se-yeast	Aluminum exposed mice	Treated with 0.1 mg kg^–1^ per day for 28 days	↓Oxidative stress; ↓Inflammatory induction from Al-induction ↓mRNA inflammatory genes in liver tissue	(97)
Se-yeast	Mouse mammary tumor (EMT6) cells xenograft mice	Treated with 912 ng Se per day for 14 days	↓MDA in lung, brain, liver, thymus, spleen and kidney. ↑ Bcl-2; ↑p53; ↓IL-4 in tumor cells	(95)
Se-yeast	Yellow broilers	Treated with 0.15 mg Se kg^–1^ per day for 8 weeks	↑TrxR1; ↑GPx1 in kidney tissue	(96)

↑, increase or upregulate; ↓, decrease or downregulate; α-SMA, alpha-smooth muscle actin; ALT, glutamic pyruvic transaminase; AST, glutamic oxaloacetic transaminase; CAT, catalase; CCl_4_, carbon tetrachloride; COX-2, cyclooxygenase-2; CXCL1, chemokine ligan-1; FBG, fast blood glucose, GSH, glutathione content; GST, glutathione S-transferases activity; GR, glutathione reductase; GPx, glutathione peroxidase activity; IFN-γ, interferon-gamma; IL-1, interleukein-1; IL-2, interleukein-2; IL-4, interleukein-4; IL-6, interleukein-6; LDL, low-density lipoproteins; MDA, malondialdehyde; MMP9, matrix metallopeptidase 9; MPC-1, monocyte chemoattractant protein-1; NF-κB, nuclear factor kappa B; Nrf2, nuclear factor erythroid 2–related factor 2; SOD, superoxide dismutase; TBAR, thiobarbituric acid reactive substances; TC, total cholesterol; TG, total triglyceride; TNF-α, tumor necrosis factor alpha; TrxR, thioredoxin reductase activity.

In comparison, Se-enriched yeast (*Saccharomyces cerevisiae*) provides antioxidant and antitumor activities in animal studies with a lower affecting dose than Se-containing plants (95, 96). Se-enriched yeast could protect from oxidative stress and increase anti-inflammation by downregulating inflammatory cytokines such as TNF-a and NF-kB in aluminum-stress mice livers (97). The bioactivity of Se-enriched yeast could be due to the presence of SeMet as the main Se compound, where its biological properties have been widely studied. Compared to Se-enriched yeast, the bioactivity of Se-enriched plants is harder to explain and conclude. Not only because of the uniqueness of Se concentration and chemical forms in different plants, but the complexity of the food matrix also plays a significant role when studying the biological properties of Se-containing plant foods (4, 98). Food matrices, including protein and carbohydrates, can incorporate with Se via biosynthesis metabolism to form complex Se structures such as selenoprotein and selenopolysaccharide. The synthesized Se molecules can play a key role in the biological activity and bioavailability of Se-enriched food in humans (99). For instance, long-chain selenopeptide synthesized in soybean showed higher resistance in gastrointestinal digestion and lower toxicity risk compared with short-chain selenopeptide (100).

## Clinical trials of selenium-enriched plant foods

Some beneficial properties of Se-enriched plant foods have been confirmed in *in vitro* cell models and *in vivo* animal studies. According to this evidence, there have been some human clinical trials performed to gain a robust understanding of the bioactivity of Se-enriched plant foods through the human metabolic system. Table 4 presents a compilation of biological properties of Se-enriched plant foods and yeast as reported in human clinical trials. Improving the activity of antioxidant enzymes in human blood systems has been discovered as the primary biological activity of Se-containing plant materials. For example, Se-containing Brazil nuts have been found to enhance GPx activities and selenoprotein P and lowering total cholesterol and LDL in older adults (101–103). Similarly, Se-enriched rice has been found to improve the total Se content and GPx activity in serum (104). Moreover, Se-enriched green onion and broccoli also showed beneficial effects in human clinical trials (105, 106). On the other hand, Se-enriched yeast has been applied as an effective and less toxic Se supplement to provide significant health properties. Se-enriched yeast could lower blood glucose, enhance insulin sensitivity, and lower the total cholesterol and LDL (107–109).

**TABLE 4:** Selenium-enriched plant foods and yeast human clinical trials.

Se-enriched food	Participants	Age group	Treatment	Functional properties	References
Onion	18 participants	50-64	50 μg Se daily for 12 weeks	↑T-cell proliferation after flu vaccination ↑IFN-γ; ↑IL-8; ↑Enzyme and perforin content in CD8 cells ↓TNF-α in CD8 cells	(105)
Broccoli	18 participants	24-65	200 μg Se per day for 3 days	↑Total Se level in plasma ↑Interleukin products in peripheral blood mononuclear cell	(106)
Rice	10 women participants	25 ± 2	80 g of Se-enriched rice (1.64 mg Se kg-1) per day for 20 days	↑Total Se level in plasma ↑GPx in plasma	(104)
Brazil nut	91 hypertensive and dyslipidaemia patients	62.1↑ ± ↑9.3	13 g of granulated Brazil nut (∼227.5 μg Se) per day for 12 weeks	↑Total Se level in plasma ↑GPx3; ↓oxidized LDL level in plasma	(102)
Brazil nut	89 dyslipidaemia and hypertensive patients	40-80	Brazil nuts 227.5 μg Se per day for 90 days	↓Total cholesterol; ↓non-HDL in serum Non-significantly different blood pressure and lipid content in serum	(181)
Brazil nut	81 hemodialysis patients	52 ± 15.2	5g Brazil nut (290.5 μg Se) per day for 3 months	↑Total Se level in plasma and erythrocyte ↑GPx in plasma	(103)
Brazil nut	61 participants	52-75	50 μg Se daily for 6 weeks	↑selenoprotein P; ↑β-catenin mRNA in blood Non-significantly decrease C-reactive protein in plasma	(101)
Se-yeast	36 polycystic ovary syndrome women	18-40	200 μg Se daily for 8 weeks	↓Cytokines IL-1; ↓TNF- α in serum ↑VEGF in serum	(182)
Se-yeast	491 participants	60-74	300 μg Se daily for 6 months and 2 years	↓Blood glucose marker hemoglobin at 6 months Non-significantly different at 2 years treatment	(107)
Se-yeast	400 participants	40-80	200 μg Se daily for 6 months	Non-significantly different in β-cell function or insulin sensitivity	(111)
Se-yeast	53 congestive heart failure patients	45-85	200 μg Se daily for 12 weeks	↑Insulin sensitivity index in serum ↓LDL; ↑HDL in serum	(108)
Se-yeast	80 lymphocytic thyroiditis patients	20-71	2 μg Se daily for 2 months, in combination with levothyroxine combined therapy	↑Therapeutic effect of levothyroxine ↓Thyroid-stimulating hormone; ↓Thyroid peroxidase antibody; ↓Thyroglobulin antibodies	(183)
Se-yeast	15 men	65-72.3	300 μg Se daily for 5 weeks	↓Epithelial-to-mesenchymal transition gene in Prostate biopsies	(184)
Se-yeast	76 participants	34.8	200 μg Se daily for 6 weeks	↓HbA1c gene refer to glycated hemoglobin in plasma Non-significantly fasten plasma glucose level	(185)
Se-yeast	60 diabetic patients	40-85	200 μg Se daily for 12 weeks	↓C-reactive protein; ↓matrix metalloproteinase-2; ↓MDA in plasma ↑Total plasma antioxidant capacity	(186)
Se-yeast	58 women with lipid profiles, plasma nitric oxide, or total antioxidant capacity conditions	18-55	200 μg Se daily for 6 weeks	↓Fasten plasma glucose level; ↓Serum insulin level; ↓Homeostasis model of assessment-insulin resistance ↓Triacylglycerol; ↑HDL level; ↑Total antioxidant capacity; ↑GSH in serum	(109)
Se-yeast	468 participants	60-74	300 μg Se daily for 5 years	↑Total Se level in plasma Non-significant different total cholesterol and HDL level in plasma	(112)
Se-enriched milk and Se-enriched yeast	20 participants	18-24	300 μg per day as Se-enriched yeast, and about 480 μg per day for Se-enriched milk for 8 weeks	Non-significantly different the plasma antioxidant enzyme	(113)

↑, increase or upregulate; ↓, decrease or downregulate; β-cell, beta-cells; CD8, cluster of differentiation-8; GPx, glutathione peroxidase activity; HbA1c, hemoglobin A1C; HDL, high-density lipoproteins; IFN-γ, interferon-gamma; IL-1, interlukein-1; IL-8, interlukein-8; LDL, low-density lipoproteins; MDA, malondialdehyde; T-cell, T-lymphocyte; TNF- α, tumor necrosis factor-alpha; VEGF, vascular endothelial growth factor.

From these findings, Se-enriched plant foods at their non-toxic concentration can deliver health benefits by increasing antioxidant activity in human serum. Daily intake of Se for humans is about 55-70 μg Se per day, with the toxic level at 400 μg Se per day. From the data in Table 4, the dose of Se-enriched plant food and Se-enriched yeast in the range of 200-300 μg Se per day could provide health benefits without showing toxic side effects (110). The information from this review suggested that Se-enriched plant foods should be a safer choice for increasing dietary Se consumption due to a moderate concentration of Se in the plant investigated, and the organic Se compounds are significantly identified in plant food materials.

Overall, not many Se-enriched plants have successfully demonstrated a significant beneficial effect in human clinical trials (111–113) compared to the amount of investigations conducted in cell-based and animal models. Many factors can affect the results of clinical trials, including genetics, age, gender, ethnicity, personal behaviors, medical conditions, etc. (114, 115), and they need to be taken into account when designing a trial. It is essential to identify the bioactive compounds present in the plant materials, study how they can influence the bioactivity of the Se-enriched plant foods and verify the bioactivity and toxicity effects of the Se-enriched plant foods from the *in vitro* human cell lines and *in vivo* animal testing. All of these will provide information on the samples’ biological properties, the corrective consumption level, and the toxicity dose of each Se-enriched plant food for human clinical trials.

## Conclusion and future prospects

The biological properties of Se-containing plant foods are closely associated with the chemical forms and concentrations of Se content in the products. The studies on Se accumulation and speciation of Se compounds could provide helpful insight into the mechanism of Se-enriched plant foods’ bioactivities. These beneficial bioactivities, including antioxidant, and anticancer properties of Se-enriched plant foods, have been positively demonstrated via *in vitro* human cell lines and *in vivo* animal studies. There is still a need for more human trials to relate the effect of Se-enriched foods and their health effects. Human clinical trials are critical to obtaining information regarding the consumption of Se-enriched food plants, considering different factors, including human genetic and age groups, and the effect of the food matrix.

Humans in different age groups (e.g., children, adults, elderly), gender, and health and physiological status (e.g., pregnancy and lactation) have different dietary requirements. Therefore, supplementing dietary Se to different groups of the population can be challenging as many factors need to be considered to ensure the supplementation deliver its intended health benefits. Due to the narrow gap between benefits and toxicity, precautions must be taken when considering Se enrichment in foods. The first thing to consider is the Se species present in the plant used for producing Se-enrichment foods. Since organic Se has far less toxicity, it is more suitable to be incorporated into food products. For safety reasons, it is essential to use Se-enriched plants that accumulate organic Se than those that accumulate a high inorganic Se content. Se-enriched plant foods with a moderate level of organic Se can be a more decent choice as a Se-supplement for all groups of people. Secondly, contamination from other metals, such as Cd and As, during Se accumulation can cause toxic stress in the plant and human health. Metal contamination in plants is mainly associated with the quality of soil and fertilizer applied during the enrichment stage. Soil quality and composition of Se fertilizer should be carefully monitored to avoid metal contamination of Se-enriched plants (116). Thirdly, limiting the consumption dose of Se-enriched food to a non-toxic level could prevent the harmful effect of Se toxicity. Regulations can be set and enforced to limit the level or serving size of Se-enriched foods to suit different groups of people. Furthermore, there is a need to establish suitable analytical methods to study Se speciation of various Se-enriched plant foods and perform more research to gather clinical information on bioactivity and toxicity when supplying Se-enriched plant food to different groups of the population. All these efforts are essential to protect from the negative effect of Se overdose, ensure safety and deliver the optimum benefit of Se-enriched foods to humans.

Future studies should cover the full spectrum of the research area, including identifying Se content and their chemical forms, in particular putting more effort on Se speciation of Se-enriched plant materials; screening their biological effects via *in vitro* assays or *in vivo* animal studies; and validating the findings in the human clinical trials. The evidence and knowledge from the above research could serve as a powerful motivation for the food industry to produce Se-enriched plant foods to combat Se deficiency and enhance life quality for the world population.

## Author contributions

PT, JX, and SQ generated the presented idea in this manuscript. PT and SQ developed the theory, scope and performed the computation of the data. PT, PS, and SQ versified, analyzed, and discussed the collected data. All authors discussed the results and contributed to the final manuscript.
