# Supplementary material for: Selenium-enriched plant foods: Selenium accumulation, speciation, and health functionality
Source: Front Nutr. 2023 Feb 6;9:962312. doi: 10.3389/fnut.2022.962312 (PMC9939470; doi:10.3389/fnut.2022.962312)
Supplement: Supplementary file 1 [file Table_1.docx]

Table S1 Selenium content in plant-based food materials.

| **Food** | **Enrichment methods** | **Total Se content (mg Se kg^-1^)** | **Selenium compounds** | | | | | | **References** |
| --- | --- | --- | --- | --- | --- | --- | --- | --- | --- |
|  |  |  | **SeMet** | **MSC** | **SeCys_2_** | **Selenite** | **Selenate** | **Other** |  |
| Wheat grains | Foliar spray with 100 g L^-1^ ha^-1^ as sodium selenate | 6.82 ± 0.93 | 67-86% |  |  |  |  |  | (Wang et al., 2020) |
| Potato | Foliar spray with 100 g L^-1^ ha^-1^ as sodium selenate | 1.04 - 1.38 | 0.7 mg Se kg^-1^ |  |  |  |  |  | (Zhang et al., 2019) |
| *Pleurotus pulmonarius* mushroom | Supply with 1 mg Se L^-1^ per day of sodium selenite for 6 days | 23.1 ± 3.4 | 13.9 mg Se kg^-1^ |  |  |  |  |  | (Milovanovic et al., 2019) |
| Pak choi | Watering with 10 μM as sodium selenate, for 168 h. | 42 | 6.46 ± 0.95 mg Se kg^-1^ |  |  |  | 17.72 ± 1.47 mg Se kg^-1^ |  | (Yu et al., 2019) |
| Wheat grains | Foliar spray with 120 g sodium selenate ha^-1^ | 2.86 | N/A |  |  |  |  |  | (Lara et al., 2019) |
| Rice grains | Foliar spray with 1L of 300 g sodium selenite or sodium selenate ha^-1^ | N/A | N/A |  |  | 35.15 mg Se kg^-1^ | 10.83 mg Se kg^-1^ |  | (Lidon et al., 2019) |
| Rice grains | Supply 1.47 mg Se kg^-1^ soil as sodium selenite | 0.32 | N/A |  |  |  |  |  | (Shen et al., 2019) |
| Rye grains | Supply 5 mg Se kg^-1^ soil as sodium selenate | 0.038 | N/A |  |  |  |  |  | (Skrypnik et al., 2019) |
| Sweet basil leaves | Supply 4 mg Se L^-1^ growth medium as sodium selenate | 31 | N/A |  |  |  |  |  | (Puccinelli et al., 2019) |
| *Cardamine violifolia* leaves | Grown in high Se region in Enshi city, China | 3700 |  |  |  |  |  | Selenolanthionine 40% | (Both et al., 2018) |
| *Cardamine enshiensis* leaves | Grown in high Se region in Enshi city, China | 2491 |  |  | 84 ± 8% | 16 ± 9% |  |  | (Cui et al., 2018) |
| Brown rice | Supply with 0.5 mg Se kg^-1^ soil as sodium selenite | 0.65 | 90% |  |  |  |  |  | (Huang et al., 2018) |
| Brown rice | Supply with 100 g Se ha^-1^ as sodium selenite | 1.69 | 4.86 μg g^-1^ in glutelin;  5.91 μg g^-1^ in prolamin |  |  |  |  |  | (Gong et al., 2018) |
| Rice grains | Supply with 5 mg Se kg^-1^ soil as sodium selenite | 6 | 2.46 mg Se kg^-1^ |  |  |  |  |  | (Gong et al., 2018) |
| Blueberry | Foliar spray with 200 mg Se L^-1^ as sodium selenate ha^-1^ | 0.095 - 0.113 |  |  |  |  |  | Organic 77% | (Li et al., 2018) |
| *Pleurotus eryngii* mushroom | Supply with 50 mg Se kg^-1^ substrate as sodium selenite | 23.06 ± 0.12 | 13.68 ± 2.69 mg Se kg^-1^ |  |  |  |  |  | (Fang et al., 2018) |
| Wheat shoot | Supply with 100 mL of 50 μM of chemosynthesis Se nanoparticle. | 3 | 1.01 mg Se kg^-1^ |  |  |  |  |  | (Hu et al., 2018) |
| Rice sprouts | Supply with 45 mg L^-1^ as sodium selenate for 10 days. | 90.15 ± 2.12 | 9.51 mg Se kg^-1^ |  |  |  | 57.2 mg Se kg^-1^ |  | (D’Amato et al., 2018) |
| Lettuce leaves | Supply with 40 μM as sodium selenate for 28 days. | 602 ± 6 | 19 mg Se kg^-1^ |  |  |  | 349.1 mg Se kg^-1^ |  | (do Nascimento da Silva et al., 2017) |
| Lion’s mane mushrooms | Supply with 100 mg Se kg^-1^ of sodium selenite for one week. | 42.3 | 20.7 mg Se kg^-1^ | 0.3 mg Se kg^-1^ |  |  |  |  | (Egressy-Molnár et al., 2016) |
| Radish | Foliar spray with 20 mg Se per plant as sodium selenate | 120 |  | 33 mg Se kg^-1^ |  |  |  |  | (Schiavon et al., 2016) |
| Carrot | Supply with 1 kg ha^-1^ as sodium selenate | 25 | 15 mg Se kg^-1^ |  |  |  |  |  | (Smoleń et al., 2016) |
| Broccoli sprout | Supply with 10 mg Se L^-1^ 250 mL per day as sodium selenate | 133 ± 12 | 7.1 ± 1.3 mg Se kg^-1^ | 68 ± 12 mg Se kg^-1^ |  |  |  |  | (Bodnar & Konieczka, 2016) |
| White mustard sprout |  | 78.0 ± 2.1 | 10.8 ± 1.9 mg Se kg^-1^ | 44.3 ± 8.0 mg Se kg^-1^ |  |  |  |  |  |
| Red cabbage sprouts |  | 82.2 ± 3.3 | 7.4 ± 1.3 mg Se kg^-1^ | 43.1 ± 7.8 mg Se kg^-1^ |  |  |  |  |  |
| Radish sprouts |  | 103.8 ± 4.4 | 2.03 ± 0.37 mg Se kg^-1^ | 12.6 ± 2.3 mg Se kg^-1^ |  |  |  |  |  |
| Daikon sprouts |  | 97.3 ± 3.8 | 2.39 ± 0.43 mg Se kg^-1^ | 16.6 ± 3.0 mg Se kg^-1^ |  |  |  |  |  |
| Alfalfa sprouts |  | 109 ± 12 | 4.80 ± 0.86 mg Se kg^-1^ | 8.6 ± 1.6 mg Se kg^-1^ |  |  |  |  |  |
| Broccolis | Supply with 700 mg Se kg^-1^ with Se-enriched *Stanleya pinnata* Pursh | 6.99 - 7.83 | 58.5% |  | 15% |  |  | GSSeSG 17% | (Bañuelos et al., 2016) |
| Carrots |  | 3.15 - 6.28 | 78.5% |  | 12% |  |  |  |  |
| Tea (*Camellia sinensis*) | Supply with 4 mg Se kg^-1^ as sodium selenite | 0.36 - 0.54 | N/A |  |  |  |  |  | (Zhao et al., 2016) |
| Tomato | Foliar spray with 1 mg Se L^-1^ as sodium selenate for 4 weeks | 0.5 ± 0.01 | N/A |  |  |  |  |  | (Zhu et al., 2016) |
| Cucumbers | Supply with 20 mg Se kg^-1^ soil as sodium selenate | 0.029 - 0.048 | N/A |  |  |  |  |  | (Businelli et al., 2015) |
| Lettuce leaves |  | 0.023 - 0.053 |  |  |  |  |  |  |  |
| Tomato |  | 0.015 - 0.020 |  |  |  |  |  |  |  |
| Broccoli heads | Foliar spray with 50 g Se ha^-1^ as sodium selenate | 1.01 ± 0.15 | 0.413 mg Se kg^-1^ | 0.12 mg Se kg^-1^ | 0.126 mg Se kg^-1^ |  |  |  | (Šindelářová et al., 2015) |
| Chickpeas | Supply with 50 μM sodium selenite for 14 days | 16.25 | 6.3 mg Se kg^-1^ |  | 39 mg Se kg^-1^ |  |  |  | (Lyubenova et al., 2015) |
| Ginger | Foliar spray with 50 mL per day of sodium selenite and sodium selenate 0.04 µg Se mL^-1^ for 4 months | 4.06 ± 1.42 | 2.04 mg Se kg^-1^. |  |  |  |  |  | (Deng et al., 2014) |
| Pakchoi | Supply with 60 mg Se L^-1^ as sodium selenate, on to the seeds at 400 mL day^-1^ for 7 days. | 311.7 ± 33.3 | 43 mg Se kg^-1^ | 70 mg Se kg^-1^ |  |  |  |  | (Thosaikham et al., 2014) |
| Broccoli sprouts | Watering with 50 μM sodium selenate per day for 7 days. | 165 |  | 90 mg Se kg^-1^ |  |  |  |  | (Ávila et al., 2014) |
| Cauliflower sprouts |  | 200 |  | 100 mg Se kg^-1^ |  |  |  |  |  |
| Green cabbage sprouts |  | 170 |  | 60 mg Se kg^-1^ |  |  |  |  |  |
| Chinese cabbage sprout |  | 280 |  | 140 mg Se kg^-1^ |  |  |  |  |  |
| Kale sprout |  | 280 |  | 120 mg Se kg^-1^ |  |  |  |  |  |
| Brussels sprout |  | 70 |  | 40 mg Se kg^-1^ |  |  |  |  |  |
| Rice | Foliar spray with organic Se onto the leaves evenly. | 0.143 |  |  |  |  |  | Inorganic 0.027 mg Se kg-1  Organic 0.116 mg Se kg-1 | (Sun et al., 2013) |
| Buck wheat seeds | Supply with 10 mg Se L^-1^ as sodium selenate, for 10 weeks. | 3.58 ± 0.80 | 2.82 ± 0.43 mg Se kg^-1^ in dehusked ripe seeds |  |  |  |  |  | (Vogrincic et al., 2009) |
| Carrots | Foliar sprayed with 100 μg Se mL^-1‑^ as sodium selenate, 8 mL once a week for 4 weeks. | 2.2 ± 0.1 | 0.4 ± 43 mg Se kg^-1^ |  |  |  |  |  | (Kápolna et al., 2009) |
| Potato | Supply with 10 mg Se L^-1^ as sodium selenate, 1.5 L day^-1^ for 55 days. | 1.10 ± 0.05 | 0.29 mg Se kg^-1^ |  |  |  | 0.33 mg kg^-1^ |  | (Cuderman et al., 2008) |
| Chive leaves | Supply with 10 mg Se L^-1^ as sodium selenate, 200 mL day^-1^ for 2 weeks. | 613 ± 5 | 3% | 52% | 44% |  |  |  | (Kápolna et al., 2007) |
| Japanese pungent radish | Supply with 500 mg Se per m^2^ as sodium selenate and sodium selenite | 32.9 ± 0.9 |  | 12.1% |  |  | 4.9% |  | (Ogra et al., 2007) |
| Green onion bulbs | Supply with 10 mg Se L^-1^ as SeMet, 200 mL per day for 2 weeks. | 14.4 ± 0.8 |  | 85% |  |  |  |  | (Kápolna & Fodor, 2006) |

**Note:** SeMet, selenomethionine; MSeMet, methyl-selenomethionine; SeCys_2_, selenocystine; GSSeSG, selenodiglutathione.

**References**

Ávila, F. W., Yang, Y., Faquin, V., Ramos, S. J., Guilherme, L. R. G., Thannhauser, T. W., & Li, L. J. F. C. (2014). Impact of selenium supply on Se-methylselenocysteine and glucosinolate accumulation in selenium-biofortified Brassica sprouts. *Food Chemistry, 165*, 578-586.

Bañuelos, G. S., Arroyo, I. S., Dangi, S. R., & Zambrano, M. C. (2016). Continued Selenium Biofortification of Carrots and Broccoli Grown in Soils Once Amended with Se-enriched S. pinnata. *Frontiers in plant science, 7*(1251). doi:10.3389/fpls.2016.01251

Bodnar, M., & Konieczka, P. (2016). Evaluation of candidate reference material obtained from selenium-enriched sprouts for the purpose of selenium speciation analysis. *LWT, 70*, 286-295. doi:<https://doi.org/10.1016/j.lwt.2016.02.016>

Both, E. B., Shao, S., Xiang, J., Jókai, Z., Yin, H., Liu, Y., Magyar, A., & Dernovics, M. (2018). Selenolanthionine is the major water-soluble selenium compound in the selenium tolerant plant Cardamine violifolia. *Biochimica et Biophysica Acta (BBA)-General Subjects*.

Businelli, D., D'Amato, R., Onofri, A., Tedeschini, E., & Tei, F. (2015). Se-enrichment of cucumber (Cucumis sativus L.), lettuce (Lactuca sativa L.) and tomato (Solanum lycopersicum L. Karst) through fortification in pre-transplanting. *Scientia Horticulturae, 197*, 697-704. doi:<https://doi.org/10.1016/j.scienta.2015.10.039>

Cuderman, P., Kreft, I., Germ, M., Kovačevič, M., & Stibilj, V. (2008). Selenium species in selenium-enriched and drought-exposed potatoes. *Journal of agricultural and food chemistry, 56*(19), 9114-9120.

Cui, L., Zhao, J., Chen, J., Zhang, W., Gao, Y., Li, B., & Li, Y.-F. (2018). Translocation and transformation of selenium in hyperaccumulator plant Cardamine enshiensis from Enshi, Hubei, China. *Plant and Soil, 425*(1), 577-588. doi:10.1007/s11104-018-3587-8

D’Amato, R., Fontanella, M. C., Falcinelli, B., Beone, G. M., Bravi, E., Marconi, O., Benincasa, P., & Businelli, D. (2018). Selenium Biofortification in Rice (Oryza sativa L.) Sprouting: Effects on Se Yield and Nutritional Traits with Focus on Phenolic Acid Profile. *Journal of agricultural and food chemistry, 66*(16), 4082-4090. doi:10.1021/acs.jafc.8b00127

Deng, B., Shen, C., Qin, X., Liang, S., & Liang, Y. (2014). Selenium speciation in ginger using capillary electrophoresis online coupled with electrothermal atomic absorption spectrometry. *Journal of analytical atomic spectrometry, 29*(10), 1889-1896.

do Nascimento da Silva, E., Aureli, F., D’Amato, M., Raggi, A., Cadore, S., & Cubadda, F. (2017). Selenium Bioaccessibility and Speciation in Selenium-Enriched Lettuce: Investigation of the Selenocompounds Liberated after in Vitro Simulated Human Digestion Using Two-Dimensional HPLC-ICP-MS. *Journal of agricultural and food chemistry, 65*(14), 3031-3038. doi:10.1021/acs.jafc.7b01188

Egressy-Molnár, O., Ouerdane, L., Győrfi, J., & Dernovics, M. (2016). Analogy in selenium enrichment and selenium speciation between selenized yeast Saccharomyces cerevisiae and Hericium erinaceus (lion's mane mushroom). *LWT-Food Science and Technology, 68*, 306-312.

Fang, Y., Zhang, Y., Wang, M., Pei, F., Xie, M., Li, P., & Hu, Q. (2018). In vitro bioaccessibility and speciation changes of selenium in Pleurotus eryngii during the growing stage. *Food & function, 9*(8), 4493-4499. doi:10.1039/C8FO00566D

Gong, R., Ai, C., Zhang, B., & Cheng, X. (2018). Effect of selenite on organic selenium speciation and selenium bioaccessibility in rice grains of two Se-enriched rice cultivars. *Food Chemistry, 264*, 443-448. doi:<https://doi.org/10.1016/j.foodchem.2018.05.066>

Hu, T., Li, H., Li, J., Zhao, G., Wu, W., Liu, L., Wang, Q., & Guo, Y. (2018). Absorption and Bio-Transformation of Selenium Nanoparticles by Wheat Seedlings (Triticum aestivum L.). *9*(597). doi:10.3389/fpls.2018.00597

Huang, G., Ding, C., Yu, X., Yang, Z., Zhang, T., & Wang, X. (2018). Characteristics of Time-Dependent Selenium Biofortification of Rice (Oryza sativa L.). *Journal of agricultural and food chemistry, 66*(47), 12490-12497. doi:10.1021/acs.jafc.8b04502

Kápolna, E., & Fodor, P. J. M. J. (2006). Speciation analysis of selenium enriched green onions (Allium fistulosum) by HPLC-ICP-MS. *84*(1-2), 56-62.

Kápolna, E., Hillestrøm, P. R., Laursen, K. H., Husted, S., & Larsen, E. H. (2009). Effect of foliar application of selenium on its uptake and speciation in carrot. *Food Chemistry, 115*(4), 1357-1363. doi:<https://doi.org/10.1016/j.foodchem.2009.01.054>

Kápolna, E., Shah, M., Caruso, J. A., & Fodor, P. (2007). Selenium speciation studies in Se-enriched chives (Allium schoenoprasum) by HPLC-ICP–MS. *Food Chemistry, 101*(4), 1398-1406. doi:<https://doi.org/10.1016/j.foodchem.2006.03.048>

Lara, T. S., Lessa, J. H. d. L., de Souza, K. R. D., Corguinha, A. P. B., Martins, F. A. D., Lopes, G., & Guilherme, L. R. G. (2019). Selenium biofortification of wheat grain via foliar application and its effect on plant metabolism. *Journal of Food Composition and Analysis, 81*, 10-18. doi:<https://doi.org/10.1016/j.jfca.2019.05.002>

Li, M., Zhao, Z., Zhou, J., Zhou, D., Chen, B., Huang, L., Zhang, Z., & Liu, X. (2018). Effects of a foliar spray of selenite or selenate at different growth stages on selenium distribution and quality of blueberries. *Journal of the Science of Food and Agriculture, 98*(12), 4700-4706. doi:10.1002/jsfa.9004

Lidon, F. C., Oliveira, K., Galhano, C., Guerra, M., Ribeiro, M. M., Pelica, J., Pataco, I., Ramalho, J. C., LeitÃO, A. E., Almeida, A. S., Campos, P. S., Ribeiro-Barros, A. I., Pais, I. P., Silva, M. M., Carvalho, M. L., Santos, J. P., Pessoa, M. F., & Reboredo, F. H. (2019). Selenium biofortification of rice through foliar application with selenite and selenate *Experimental Agriculture, 55*(4), 528-542. doi:10.1017/S0014479718000157

Lyubenova, L., Sabodash, X., Schröder, P., & Michalke, B. (2015). Selenium species in the roots and shoots of chickpea plants treated with different concentrations of sodium selenite. *Environmental Science Pollution Research, 22*(21), 16978-16986.

Milovanovic, I., Lajin, B., Braeuer, S., Steiner, O., Lisa, F., & Goessler, W. (2019). Simultaneous selenium and sulfur speciation analysis in cultivated Pleurotus pulmonarius mushroom. *Food Chemistry, 279*, 231-236. doi:<https://doi.org/10.1016/j.foodchem.2018.12.009>

Ogra, Y., Kitaguchi, T., Ishiwata, K., Suzuki, N., Iwashita, Y., & Suzuki, K. T. (2007). Identification of selenohomolanthionine in selenium-enriched Japanese pungent radish. *Journal of analytical atomic spectrometry, 22*(11), 1390-1396.

Puccinelli, M., Malorgio, F., Maggini, R., Rosellini, I., & Pezzarossa, B. (2019). *Biofortification of Ocimum basilicum L. plants with selenium*.

Schiavon, M., Berto, C., Malagoli, M., Trentin, A., Sambo, P., Dall'Acqua, S., & Pilon-Smits, E. A. H. (2016). Selenium Biofortification in Radish Enhances Nutritional Quality via Accumulation of Methyl-Selenocysteine and Promotion of Transcripts and Metabolites Related to Glucosinolates, Phenolics, and Amino Acids. *Frontiers in plant science, 7*(1371). doi:10.3389/fpls.2016.01371

Shen, J., Jiang, C., Yan, Y., & Zu, C. (2019). Selenium Distribution and Translocation in Rice (Oryza sativa L.) under Different Naturally Seleniferous Soils. *11*(2), 520.

Šindelářová, K., Száková, J., Tremlová, J., Mestek, O., Praus, L., Kaňa, A., Najmanová, J., & Tlustoš, P. (2015). The response of broccoli (*Brassica oleracea*) varieties on foliar application of selenium: uptake, translocation, and speciation. *Food Additives Contaminants: Part A, 32*(12), 2027-2038.

Skrypnik, L., Kurkova, T., & Chupakhina, G. (2019). Accumulation of selenium in rye plants (*Secale cereale L.*) at different stages of development and grain quality due to selenate soil supplementation. *Applied Ecology and Environmental Research, 17*(2), 2385-2421.

Smoleń, S., Skoczylas, Ł., Ledwożyw-Smoleń, I., Rakoczy, R., Kopeć, A., Piątkowska, E., Bieżanowska-Kopeć, R., Koronowicz, A., & Kapusta-Duch, J. (2016). Biofortification of Carrot (Daucus carota L.) with Iodine and Selenium in a Field Experiment. *Frontiers in plant science, 7*(730). doi:10.3389/fpls.2016.00730

Sun, M., Liu, G., & Wu, Q. (2013). Speciation of organic and inorganic selenium in selenium-enriched rice by graphite furnace atomic absorption spectrometry after cloud point extraction. *Food Chemistry, 141*(1), 66-71.

Vogrincic, M., Cuderman, P., Kreft, I., & Stibilj, V. (2009). Selenium and its species distribution in above-ground plant parts of selenium enriched buckwheat (Fagopyrum esculentum Moench). *Analytical Sciences, 25*(11), 1357-1363.

Wang, M., Ali, F., Wang, M., Dinh, Q. T., Zhou, F., Bañuelos, G. S., & Liang, D. (2020). Understanding boosting selenium accumulation in Wheat (Triticum aestivum L.) following foliar selenium application at different stages, forms, and doses. *Environmental Science and Pollution Research, 27*(1), 717-728. doi:10.1007/s11356-019-06914-0

Yu, Y., Liu, Z., Luo, L.-y., Fu, P.-n., Wang, Q., & Li, H.-f. (2019). Selenium Uptake and Biotransformation in Brassica rapa Supplied with Selenite and Selenate: A Hydroponic Work with HPLC Speciation and RNA-Sequencing. *Journal of agricultural and food chemistry, 67*(45), 12408-12418. doi:10.1021/acs.jafc.9b05359

Zhang, H., Zhao, Z., Zhang, X., Zhang, W., Huang, L., Zhang, Z., Yuan, L., & Liu, X. (2019). Effects of foliar application of selenate and selenite at different growth stages on Selenium accumulation and speciation in potato (Solanum tuberosum L.). *Food Chemistry, 286*, 550-556.

Zhao, H., Huang, J., Li, Y., Song, X., Luo, J., Yu, Z., & Ni, D. (2016). Natural variation of selenium concentration in diverse tea plant (Camellia sinensis) accessions at seedling stage. *Scientia Horticulturae, 198*, 163-169. doi:<https://doi.org/10.1016/j.scienta.2015.11.026>

Zhu, Z., Chen, Y., Zhang, X., & Li, M. (2016). Effect of foliar treatment of sodium selenate on postharvest decay and quality of tomato fruits. *Scientia Horticulturae, 198*, 304-310. doi:<https://doi.org/10.1016/j.scienta.2015.12.002>
